# The effects of six sprint interval training sessions on muscle oxygenation and swimming performance in untrained swimmers

**DOI:** 10.3389/fspor.2024.1451738

**Published:** 2024-10-31

**Authors:** Athanasios A. Dalamitros, Dimitrios Tzivanis, Alexandra Martín-Rodríguez, Eleni Semaltianou, Georgios Mavridis, Vasiliki Manou

**Affiliations:** ^1^ Laboratory of Evaluation of Human Biological Performance, School of Physical Education & Sport Sciences, Aristotle University of Thessaloniki, Thessaloniki, Greece; ^2^ Faculty of Sports Sciences, Universidad Europea de Madrid, Madrid, Spain; ^3^ Faculty of Applied Social Sciences and Communications, UNIE, Madrid, Spain

**Keywords:** sprint intervals, muscle oximetry, short training period, field testing, swimming

## Abstract

The current study examined the changes in muscle oxygenation values and swimming performance after six sessions of sprint interval training during a three-week period in untrained swimmers. Twelve swimmers of both genders (age: 23.5 ± 5.6yrs) executed the twice-weekly experimental training protocol (EXP, *n* = 12), consisting of a 4 × 50 m front-crawl swimming (repeated sprint training—RST) with maximal intensity, and 2 min of passive recovery in between, after a short in-water warm-up. The control group (CON, *n* = 9) performed a continuous swimming set (200 m) at 120 b pm^−1^, with the same weekly frequency. Performance times in two maximum swim trials (400 m: T400 and 50 m: T50), muscle oxygenation of the deltoid muscle (SmO_2_) immediately after T400 and T50, 1-min heart rate recovery (HRR1) after T400, T50, and swim strokes during both swim trials (S/T400, S/T50) were assessed. For the EXP group, T400 improved by 2.4 (*p* = 0.011). In contrast, T50 presented no significant improvement (1%, *p* > 0.05). SmO_2_ decreased at T400 (5.5%, *p* = 0.017) and increased at T50 (3.7%, *p* = 0.030). HRR1 improved after T400 (7.9%, *p* = 0.002), T50 (4.6%, *p* = 0.005) and RST (9.6%, *p* = 0.002). S/T400 and S/T50 remained relatively unchanged (*p* > 0.05). The CON group presented no significant changes in any of the variables examined. In conclusion, six sprint interval training sessions can improve aerobic capacity over a 3-week training period, as indicated by the enhanced T400 performance and the reduced HRR1 values, in previously trained swimmers. Finally, the sensitivity of the near-infrared spectroscopy method to detect short-term training-induced changes is highlighted.

## Introduction

Sprint interval training (SIT), a form of high-intensity interval training performed at the severe-intensity domain, has been shown to significantly improve endurance sports performance in trained athletes of different individual sports such as running (1), cycling (2), and triathlon (3). SIT protocols are based on executing repetitions between 15 s and 2 min of duration with interval rests at low intensity or rest, including a variety of work-to-rest ratios. Since, as well before, the pioneering work by Gibala et al. (4), substantial interest has been focused on analyzing the effect of short intervention periods of SIT protocols (5), typically lasting 2–3 weeks, on time-trial performance, markers of endurance performance (e.g., VO_2_max), and enzyme activity (e.g., glycogen concentration) in untrained individuals (6). According to a review and meta-analysis by Hall et al. (7), studies analyzing the effect of short-term SIT protocols on physical performance measures in healthy adult individuals typically demonstrate medium improvements, with the authors suggesting that a restricted range of outcomes corresponding to the research hypothesis should be considered in future related studies.

In swimming, SIT sets are integrated by coaches into the training regimen of athletes to induce physiological and performance adaptations (8). In the study of Bielec et al. (9), the execution of six SIT sessions resulted in enhanced swimming velocity during a 25 m swim test and increased power outputs during a Wingate test, even though no notable enhancement in kinematic variables during an eight- 25-m repetition protocol was detected. Arsoniadis and colleagues (10) demonstrated that the critical speed of competitive swimmers increased following 6 weeks of SIT; however, in the same study, performance during a 4 × 50 m, and 100 m tests and BLa concentration did not significantly alter. Nonetheless, there is a lack of data indicating the significance of short-term SIT sets in enhancing swimming performance during competitive distances.

Real-time data collection of muscle oxygenation (O_2_) saturation (SmO_2_) kinetics using near-infrared spectroscopy (NIRS) has been outlined as a valuable and relatively low-cost tool for comprehensively analyzing skeletal muscle's physiological function and adaptations, as well as assessing the level of internal exertion during exercise training (11). This technology has been applied in swimming as a complementary method to BLa and heart rate (HR) testing procedures to analyze acute training responses. In this context, Dalamitros et al. (12) specified that following maximal swimming protocols that included ultra-brief (15 m), and brief (25 m) interval efforts, a strong correlation was observed between SmO_2_, HR, and BLa values. This correlation was compared to the results obtained after a similar protocol with initially lower intensity interval efforts. NIRS has also been used to analyze acute training responses in swimmers of different competitive levels (13). As Morouço et al. (14) reported, SmO_2_ values of the deltoid muscle during front-crawl swimming accurately depict the equilibrium between oxygen delivery and extraction in the body's primary muscle group responsible for both horizontal propulsion and the recovery phase, presenting high accessibility during data collection (15).

Considering the above, a training study examining the effect of a low-volume SIT on different competitive swimming distances, including the measurement of muscle oxygenation kinetics, may enhance the current knowledge of this training approach and accentuate the potential benefits of using muscle oximetry in swimming. Therefore, this study aimed to assess the effect of six sprint interval training sessions over 3 weeks on 400 m, 50 m, and repeated sprint swimming performance and muscle oxygenation values in previously trained swimmers. The following hypotheses were proposed: (i) the execution of the 3-week SIT protocol will result in improvement during the “aerobic” swimming test in previously trained swimmers, and (ii) muscle oxygenation values will alter, following different responses, during the short and longer swimming tests.

## Materials and methods

### Subjects

Based on the findings of a previous study (16), the power analysis conducted using the G_*_Power software package, determined that a sample size of 12 participants would provide 95% power for detecting meaningful effects, with a statistical significance of 0.05. Finally, a total of 21 untrained swimmers, with a competitive background primarily focused on front-crawl and specializing in sprint events, participated voluntarily in this study after providing written informed consent. Twelve of the participants were randomly assigned to the experimental group (EXP: 9 males & 3 females, 83.0 ± 14.9 kg, 23.5 ± 2.6 years, 183.0 ± 7.5 cm), while the rest were allocated to the control group (CON: 5 males & 4 females, 75.7 ± 17.4 kg, 23.1 ± 2.2 years, 178.2 ± 6.6 cm). All participants refrained from any swimming-related training period exceeding 6 months and high-intensity exercise during the testing and training period. The testing procedures complied with the Helsinki Declaration and received approval from the Research Ethics Committee (approval number 126/2022).

### Procedures

**Figure 1:**
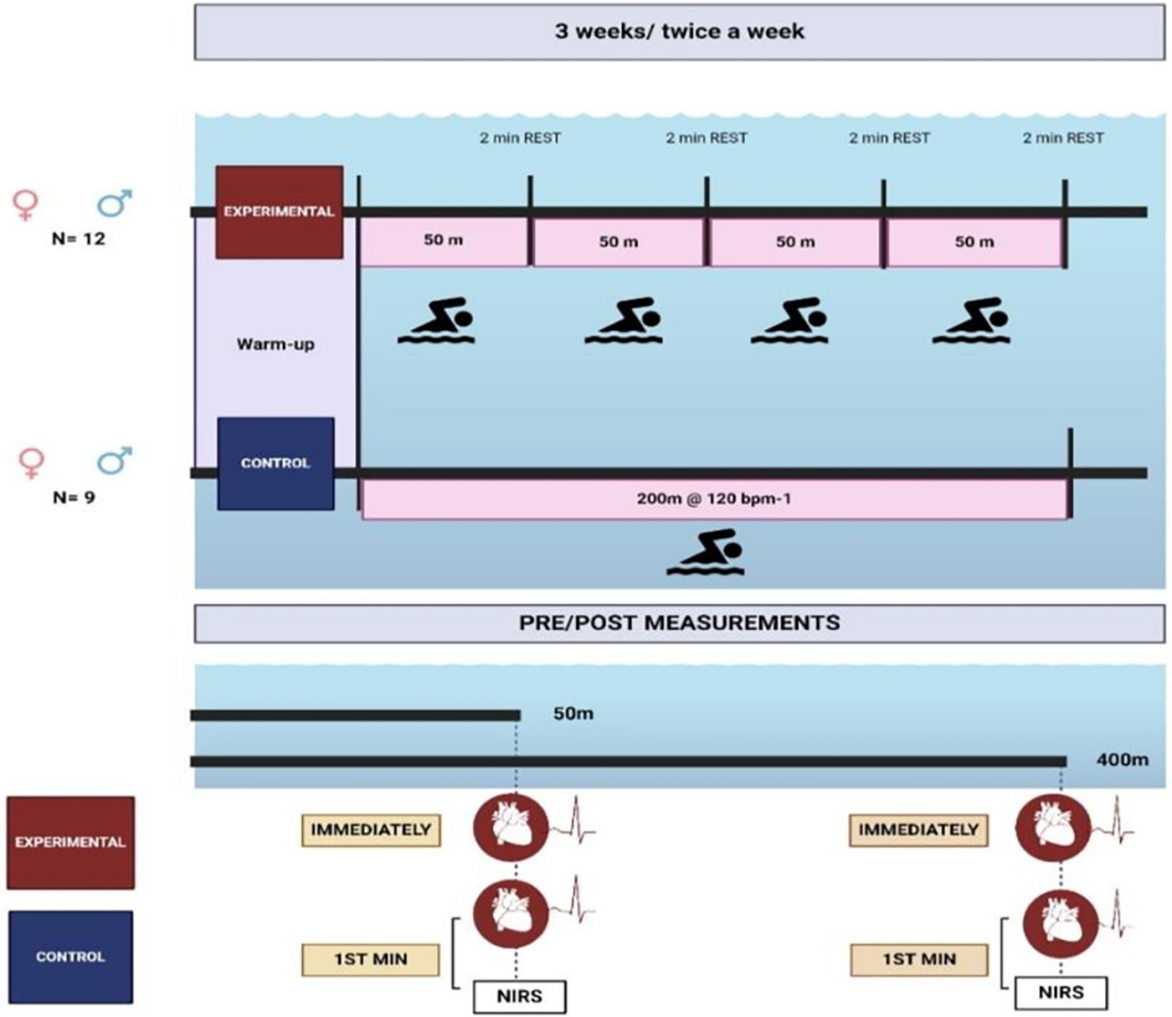
Schematic representation of the experimental procedure.

In this study, a randomized controlled pre-post design was used. After a familiarization session with the testing protocol (conducted the week before initiation of the study), two testing sessions were employed 2 days before (T1: pre) and 2 days after the 3 weeks (T2: post) of either the SIT or the CON condition. During the first testing session, all participants completed a brief questionnaire specifying their date of birth, body weight and height, best swimming performance, and years of training experience. In this session, the skinfold thickness was also measured. After a standardized warm-up of 600 m (400 m continuous swimming/2 × 25 m arm and kick drills/50 m progressive/2 × 25 m sprints/50 m cooldown), and following a 2-min passive rest, all participants executed a 400 m (T400) and a 50 m (T50) maximal swim tests, with a 30 min interval between. Following the same warm-up previously described, the participants of the EXP group performed the SIT protocol consisting of 4 × 50 m, intercepted with a 2-min passive rest, at maximal intensity. During the same 3-week period, the CON group applied an identical warm-up, followed by a 200 m self-paced easy swim. In this case, the intensity during swimming was set at approximately 120 b m^−1^, as established by a preliminary test, recorded every 50 m using a heart rate monitor. The participants were evaluated in an indoor 25 m swimming pool with a temperature of 26–27°C, with all measurements and training sessions taking place from 9:30 to 11:00 in the morning, during the same period (March). All swim tests and training sessions were performed with the front-crawl technique, starting with a push-off start from within the water. Participants were instructed to avoid underwater gliding and advised to follow the same diet, hydration, and sleeping habits the day before each test. Participants in the EXP group received performance time feedback after each 50 m maximum effort during the SIT protocol. The experimental procedure is summarized in Figure 1.

### Instrumentation

Near-infrared spectroscopy (NIRS), using a MOXY portable device (MOXY, Fortiori Design LLC, Hutchinson, Minnesota, USA), was employed to measure SmO_2_ values. This device was placed on the participants’ middle portion of the dominant arm's deltoid muscle, aligned with the direction of the specific muscle fibers. Its location was marked with a permanent marker to ensure consistency during each measurement. The SmO_2_ levels were measured while seated by the same experienced examiners, with the arms hanging naturally at the sides in a fully relaxed position (12). Subsequent SmO2 data were collected during the first minute at rest, allowing sufficient time for exiting the water. The device's receiving optodes were cleaned during each measurement to ensure reliability. All participants had skinfold thickness below the accepted threshold of 12 mm at the measurement side, as previously suggested (17). A Harpenden skinfold caliper (Creative Health, Dallas, USA) was used for this measurement. The Polar S610 heart rate monitor was used to measure exercise heart rate immediately after each test and during the first minute of the recovery phase (HRR1). The time for each 400 m (T400) and 50 m (T50) test, as well as during the 4 × 50 m (repeated sprint performance: RST) of the SIT protocol, was recorded using a digital stopwatch (FINIS 3X300, Finis Inc., Livermore, CA, USA) by two independent timekeepers. Swim strokes were recorded on the 3rd, 7th, 11th, and 15th 25 m during the T400 (S/T400), and on the 2nd 25 m for the T50 m (S/T50), by two research collaborators.

### Statistical analysis

Statistical analyses were performed using the SPSS statistical package (v. 28.0). Values were calculated for swimming performance, kinematics, and physiological parameters during both testing points (T1 & T2) presented as means ± SD. Between-participant data (age, body height, weight, swimming trial performance for the 50 m & 400 m tests) were tested for the assumption of homogeneity of variances with Levene's test. Paired samples *T*-test with the Bonferroni correction was used to analyze the difference between the two groups in T1 and T2, for all the parameters analyzed (T400 & T50, RST, swimming strokes at T400 [S/T400] and T50 [S/T50], SmO_2,_ and HRR1 during T400, T50, and RST). Cohen's d effect sizes (ES) and 90% confidence intervals were also calculated for differences between T1 and T2 testing points for training-related changes in both groups. Effect sizes (ES) of <0.19, 0.20–0.59, 0.60–1.19, 1.20–1.99, and 2.0–4.0 were considered trivial, small, moderate, large, and very large differences, respectively (18). Statistical significance was set at *a* = 0.05.

## Results

**Figure 2:**
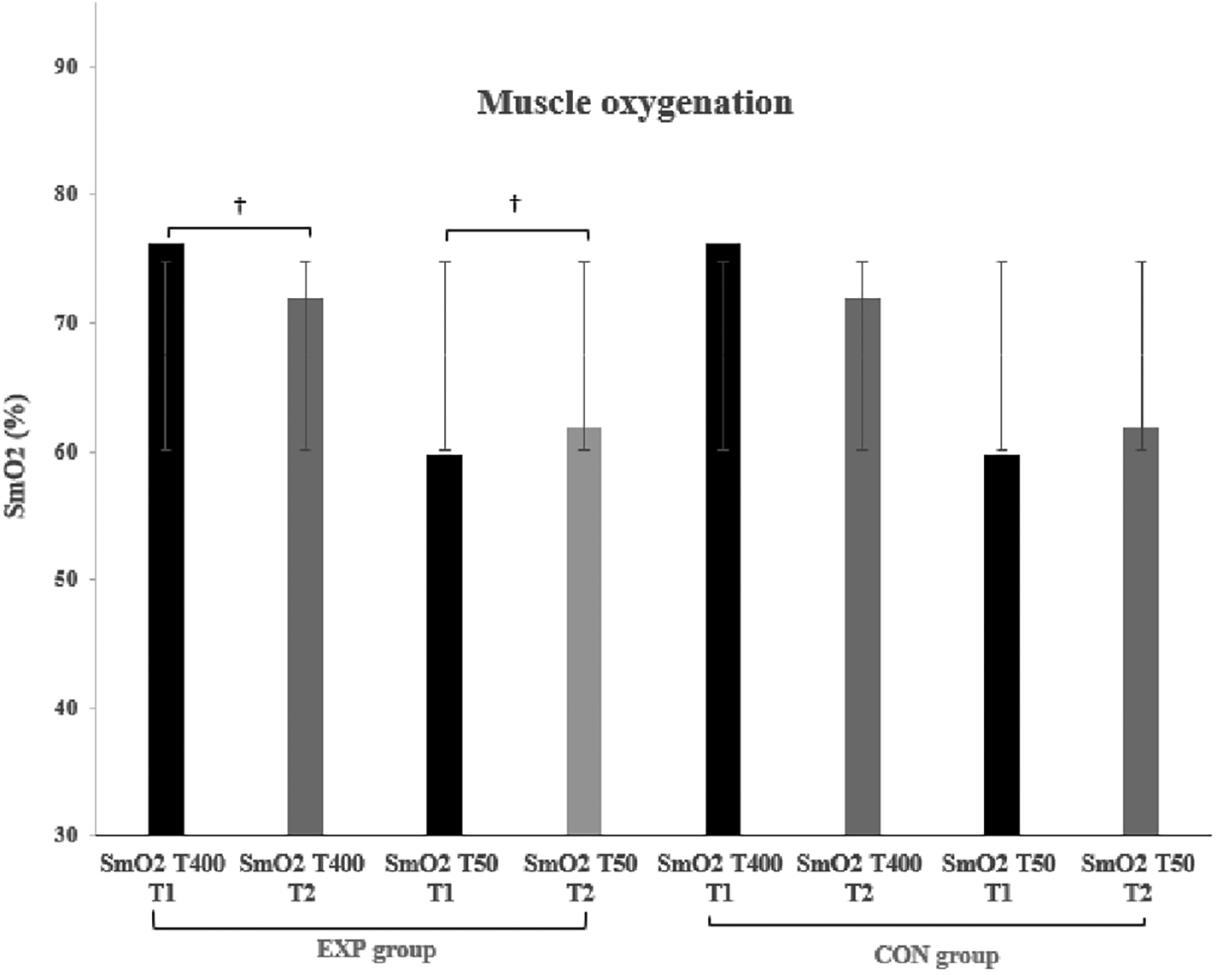
Muscle oxygenation values in both groups (EXP & CON), time trials (400 & 50 m), and testing points (T1 & T2). ^†^*p* = <0.05. Data are presented as mean ± SD.

**Figure 3:**
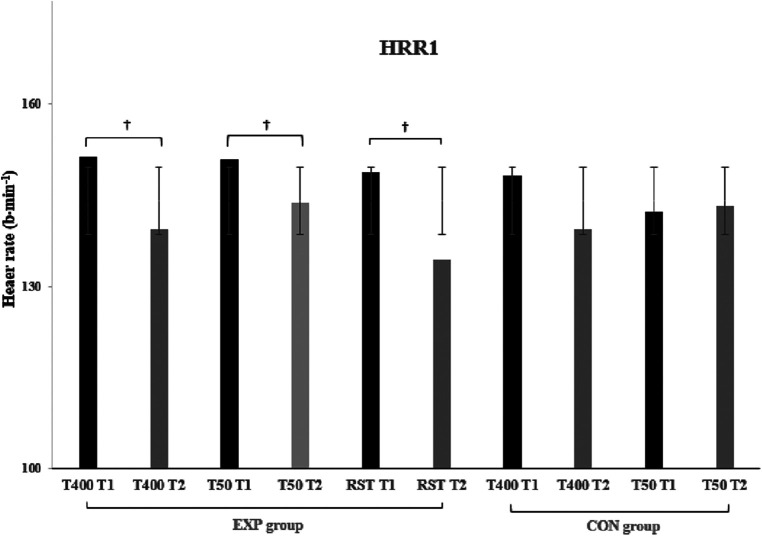
Heart rate recovery during the 1st post-exercise minute in both groups (EXP & CON), time trials (400 & 50 m), and testing points (T1 & T2). ^†^*p* < 0.05. Data are presented as mean ± SD.

Paired samples *T*-test for the EXP group, showed significant improvement in T400 and RST between T1 and T2 (*t* = 3.044 and 2.571, *p* = 0.011 and 0.026, ES = 0.879 and 0.742, respectively) (Figure 2). In contrast, T50, S/T400, and S/T50 remained relatively unchanged after the six training sessions (*p* > 0.05). SmO_2_ values were significantly reduced at T400 (*t* = 2.816, *p* = 0.017, ES = 0.813) and increased at T50 (*t* = −2.485, *p* = 0.030, ES = −7.17) at T2. HRR1 data in all swim tests (T400, T50, and RST) were significantly reduced at T2 (*t* = 2.374–4.035, *p* = 0.002–0.037) (Figure 3). Finally, the CON group showed no significant differences in any of the parameters tested in T2. Table 1 demonstrates the mean ± SD values for performance times, SmO_2_, swim strokes, and the percentage changes in the EXP and CON groups during both testing points.

**Table 1:** Mean ± SD values and percentage changes for swimming performance, swimming strokes, and physiological parameters in both groups (EXP & CON) and testing periods (T1 and T2).

Variable	EXP group			CON group		
	TP_1_	TP_2_	% Change	TP_1_	TP_2_	% Change
T400 m (s)	327.0 ± 40.2	319.1 ± 36.9	2.4*	395.9 ± 85.6	392.6 ± 76.1	0.8
T50 m (s)	28.6 ± 3.8	28.3 ± 3.7	1.0	31.9 ± 6.8	31.6 ± 6.3	0.9
–RST (s)	31.0 ± 3.7	30.2 ± 3.4	2.6*	–	–	–
RST HRR1 (b·min^−1^)	148.8 ± 18.6	134.4 ± 16.3	9.6*	–	–	–
SmO_2_ T400 (%)	76.2 ± 8.8	72.0 ± 6.7	5.5*	77.0 ± 12.4	76.9 ± 12.1	0.1
SmO_2_ T50 (%)	59.7 ± 9.2	61.9 ± 10.3	3.7*	70.3 ± 15.1	69.8 ± 13.9	0.7
T400 HRR1 (b·min^−1^)	151.3 ± 23.2	139.4 ± 18.0	7.9*	138.1 ± 12.7	136.6 ± 11.9	1.1
T50 HRR1 (b·min^−1^)	150.8 ± 17.8	143.8 ± 18.5	4.6*	142.2 ± 7.5	143.3 ± 7.6	0.8
S/T400 (*n*)	15.9 ± 2.9	15.7 ± 2.5	1.3	22.1 ± 6.3	22.6 ± 6.3	1.8
S/T50 (*n*)	18.9 ± 2.8	19.1 ± 2.8	1.1	23.0 ± 6.1	23.2 ± 5.7	0.9

* Significance at *p* < 0.05, T400 = maximum 400 m swim trial, T50 = maximum 50 m swim trial, RST = repeated sprint training set (4 × 50 m), HRR1 = heart rate recovery at the 1st-minute post-exercise, S/T400 = mean strokes for every 4th 25 m during T400 and S/T450 = total strokes for the 2nd 25 m during T50.

## Discussion

The objective of this study was to examine the changes in swimming performance in two competitive distances, one widely used to assess swimmers’ aerobic profile (400 m) (19), and one expressing anaerobic power (50 m) (20), as well as muscle oxygenation values after six sessions of sprint interval training performed over a 3-week period in untrained swimmers. Additionally, to evaluate other important training variables, assessed by swim coaches during daily practice, we included the measurement of post-exercise heart rate recovery, performance during the sprint interval set, and swimming stroke count. Following our research hypothesis, we found that six high-intensity interval-type workouts improved performance in an aerobic-oriented test (400 m), and modified muscle oxygenation values measured during swimming tests of different competitive distances.

To our knowledge, this is the first study analyzing the effect of a short SIT period on swimming performance depending on both the anaerobic and aerobic energy systems assessed during field testing. The training protocol applied in this study, characterized by specific duration (6 weeks), volume (four sets), and rest intervals (4 min), in two competitive swimming distances in untrained individuals, was tailored to the research objectives. Additionally, this study included untrained athletes, as short-term training protocols will likely have minimal effects on highly competitive swimmers. Consequently, the results presented here may not be directly comparable with those of other studies that employed different training regimens in individual sports. Earlier studies utilizing similar SIT protocols in untrained young male and female individuals have typically evaluated mean and peak power output during incremental protocols (7). As an exception, Hazell et al. (21) reported improvements of 5.2% and 9.5% during a 5-km time trial (covered in approximately 9.5 min) and the 30-s Wingate anaerobic test, respectively.

A comprehensive understanding of the physiological processes that occur in muscles during dynamic exercise is crucial for accurately assessing the level of internal load (11). Upon analyzing short-term changes in muscle oxygenation during repeated sprint training protocols, significant information can be derived regarding the application of muscle oximetry in sports science (22). For instance, research on kayak athletes indicated that implementing SIT protocols over 4 weeks of approximately 30 and 40-s all-out efforts leads to specific and significant peripheral adaptations (increase in maximal muscle deoxygenation) in response to the training stimuli (23). Another study measuring SmO_2_ values highlights this method to detect peripheral muscle oxygenation changes after an additional 6-week SIT protocol (applied once weekly) and positive performance adaptations during an intermittent fitness test in elite female hockey players (24). More recent data on collegiate swimmers suggested that 5 weeks of a specific dry-land protocol leads to enhanced 100 m performance accompanied by notable local SmO2 adaptations (25).

While interpreting our study's results, we should consider the significant differences in the physiological and kinematic profiles of the T400 and T50 tests. Thus, we can propose that participants in the EXP group improved their ability to sustain effort after a high-intensity, continuous aerobic-type effort. In other words, the EXP group showed enhanced skeletal muscle capacity for oxygen delivery and utilization, and/or mitochondrial function (26). Along with the finding of reduced post-exercise HR recovery, indicating aerobic adaptations (27), and improved performance during the T400 time-trial, a testing procedure characterized by a significant contribution of aerobic metabolism (28), we can suggest an elevated aerobic capacity achieved after a short period (3 weeks) in untrained athletes. Moreover, the positive change obtained in swimming performance during the RST (expressed as the mean 50 m performance time) can be attributed to the significant contribution (and improvement) of the aerobic metabolism characterizing this training method (29, 30), indicating greater fatigue resistance and faster phosphocreatine resynthesis, as previous studies reported (31, 32), along with the adequate rest interval applied here (i.e., work to rest ratio of 1:4). This is realized even though T400 and RST represent different metabolic pathways (i.e., predominantly aerobic energy turnover vs. anaerobic metabolism), reinforcing the effectiveness of the SIT method to produce aerobic-type adaptations, with a reduced time commitment, as earlier mentioned (33).

The opposite results were demonstrated for the T50 test after the six training sessions; that is, increased SmO_2_ values following a short sprint effort possibly expressed as an enhanced ability to sustain maximal intensity efforts, improved microvascular perfusion (34), and, probably, an increased functional oxidative capacity that accompanies high-intensity efforts, even during a short training period (31). This change, importantly, did not come with an improved sprint performance. Thus, the total duration of the training stimuli was insufficient to induce performance-related changes. Therefore, incorporating more extended-duration interventions and including progression during the SIT protocols applied, while, in parallel, adding strength training or plyometrics can probably be considered an effective strategy when the target is to improve sprint performance (7, 28), along with SIT, in swimmers. It should be noted that immediately after the completion of T400 and T50, HR values were almost identical, suggesting a similar level of effort during both testing points in the EXP group (169.2 ± 10.1 vs. 167.4 ± 9.2, *p* > 0.05, and 167.4 ± 10.3 vs. 165.8 ± 10.4, *p* > 0.05, for the T400 and T50 tests, during T1 and T2, respectively). The same was also observed for the CON group (166.2 ± 10.3 vs. 165.4 ± 7.0, *p* > 0.05, and 161.6 ± 9.7 vs. 162.7 ± 9.2, *p* > 0.05, for the T400 and T50 tests, during T1 and T2, respectively).

This study has certain strengths such as including a control group and a familiarization session before conducting any testing procedure, along with evaluating different competitive distances under field conditions. However, some limitations need to be recognized. First, adding BLa measurements would probably provide a more accurate evaluation of the internal load and the metabolic responses during the testing procedures. Nevertheless, as previously reported, SmO_2_ and BLa values show correlated responses after short swimming efforts (12). Second, no real-time data was received to avoid any movements of the NIRS device during the maximal arm stroke actions. Instead, we evaluated 1-min post-test values, a methodology perceived as more practical during actual training conditions. Finally, we chose not to include the RST testing procedure in the CON group, since its training period included moderate effort; thus, no significant alterations in swimming performance would be expected.

In conclusion, engaging in six sprint interval training sessions over a 3-week training period can lead to enhanced aerobic adaptations and repeated sprint performance in untrained swimmers. In contrast, maximal swimming velocity and anaerobic performance, expressed by the 50 m swim test, remained unchanged. This study also emphasizes the sensitivity of the NIRS method to detect training-induced alterations in muscle oxygenation over a short-term timeframe, offering an enhanced understanding of skeletal muscle patterns during competitive distances relying on different energy systems. From a practical standpoint, swim coaches and researchers can leverage the results presented here to gain valuable insights into swimmers’ physiological responses. Hence, integrating muscle oxygenation measurements into training programs may allow coaches to inform training decisions and develop training protocols, for instance, in athletes after long training cessation periods or for individuals who return to sport after extended periods of inactivity, as in the case of master athletes.

## Data Availability

The raw data supporting the conclusions of this article will be made available by the authors, without undue reservation.
